# Hydrogenolysis of glycerol over TiO_2_-supported Pt-WO_x_ catalysts: Effects of the TiO_2_ crystal phase and WO_x_ loading

**DOI:** 10.3389/fchem.2022.1004925

**Published:** 2022-09-21

**Authors:** Yaju Wang, Zhiming Zhou, Chao Wang, Leihong Zhao, Qineng Xia

**Affiliations:** ^1^ Key Laboratory of the Ministry of Education for Advanced Catalysis Materials, Institute of Physical Chemistry, Zhejiang Normal University, Jinhua, China; ^2^ College of Biological, Chemical Science and Engineering, Jiaxing University, Jiaxing, China; ^3^ College of Chemical Engineering, Qingdao University of Science and Technology, Qingdao, China; ^4^ Yankuang Technology Co., Ltd., Shandong Energy Group Co., Ltd., Jinan, China

**Keywords:** glycerol, hydrogenolysis, 1,3-propanediol, Pt-WO x based catalyst, TiO 2, crystal phase

## Abstract

The selective hydrogenolysis of glycerol to 1,3-propanediol (1,3-PDO) with high added value is attraction but challenging. Pt-WO_x_-based catalysts have been extensively studied in the selective hydrogenolysis of glycerol. The catalyst support and the physicochemical state of WO_x_ play important roles on this reaction. In this paper, Pt-WO_x_ catalysts supported on TiO_2_ with different crystal forms were prepared and studied for their catalytic performance in hydrogenolysis of glycerol. It was observed that the catalytic performance of anatase-type (A-type) TiO_2_-supported catalyst (Pt/W/A-Ti) is much better than that of the rutile-type (R-type) TiO_2_ catalyst (Pt/W/R-Ti) due to its higher stability. Furthermore, the influence of W loading amount and state were thoroughly investigated for the Pt/W/A-Ti catalysts, and Pt/W/A-TiO_2_ with 5 wt% loading of WO_x_ achieved the best catalytic performance (100% conversion of glycerol and 41% yield of 1,3-PDO under the optimal reaction conditions), owing to the suitable WO_x_ domains and high dispersion of W species, as evidenced by XRD patterns and TEM images. Mechanism study by *in-situ* DRIFTS experiments indicated that glycerol was first converted to 3-hydroxypropanal and then converted to 1,3-PDO through subsequent reactions.

## Table of Content

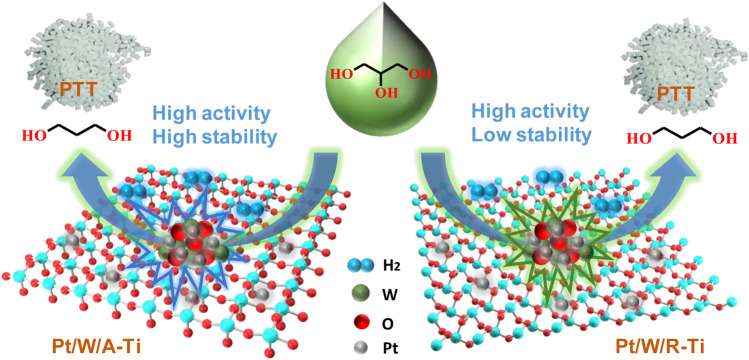

## Introduction

Biomass energy is currently receiving immense attention as a renewable energy source (ArthurRagauskas et al., 2006) (Pan et al., 2022). The production of glycerol is primarily associated with the process of biodiesel refining. Biodiesel refining of approximately 10 tons results in the production of approximately 1 ton of glycerol (Xu et al., 2021). Conversion of excess glycerol to value-added chemicals such as 1, 2- Propanediol (Dieuzeide et al., 2016), 1, 3- Propanediol (1,3-PDO) (Cheng et al., 2021), dihydroxyacetone (M. Walgode et al., 2021), acrolein (Talebian-Kiakalaieh & Amin, 2017) and lactic acid (Arcanjo et al., 2019) has attracted extensive attention in recent years (Tadahiro Kurosaka et al., 2008). Among them, 1,3-PDO is an important bulk chemical feedstock which is industrially used to synthesize polytrimethylene terephthalate (PTT), polyurethanes, and cyclic compounds (Alvise Perosa, 2005). Besides, 1,3-PDO imparts various properties such as light stability, improved elasticity, and biodegradability to polymers (Julien Chaminand et al., 2004).

Efficient conversion of glycerol to 1,3-PDO through selective hydrogenolysis is attraction but challenging. Among the numerous catalysts studied so far, Ir-Re (Yoshinao Nakagawa et al., 2010) (Yasushi Amada et al., 2011) (Deng et al., 2015) (Liu et al., 2019) and Pt-W (Shi et al., 2018) (Jarauta-Córdoba et al., 2021) (Zhu et al., 2015) (Liu et al., 2020) bifunctional catalysts have been reported to exhibit the most promising performance. In particular, Pt-W-based catalysts are more durable and cost-competitive than Ir-Re-based catalysts. These catalysts have potential practical applications. Nonetheless, the activity and selectivity of the Pt-W catalysts seem to be lower than those of the Ir-Re catalysts, so there is an urgent need for the development of modification methods to improve the productivity of 1,3-PDO. In most modification studies, it is quite common to change the support to obtain highly active catalysts. Common supports such as ZrO_2_ (Zhou et al., 2016), Al_2_O_3_ (Jarauta-Córdoba et al., 2021), SiO_2_ (Zhou et al., 2020), TiO_2_ (Zhang et al., 2013), etc., have been used to conduct the experiments. These supports can improve the activity of the Pt-W catalyst to a certain extent.

Among the above carriers, TiO_2_ has become an excellent catalyst carrier attracting much attention due to its excellent chemical stability, non-toxicity and low cost (Xiaojie Zhang et al., 2017). Therefore, we introduced TiO_2_ as a support into a Pt/W-based catalyst for the hydrogenolysis of glycerol. Before us, Zhang et al. (2013) prepared a Pt/W_n_-Ti_(100-n)_ catalyst using evaporation-induced self-assembly (EISA) strategy for hydrogenolysis of glycerol to 1,3-PDO, and 14.9% yield of 1,3-PDO was obtained. Herein, we prepared two TiO_2_-supported Pt-WO_x_ catalysts with different crystal phases (anatase type and rutile type) and WO_x_ loading, and studied the effects of TiO_2_ crystal phases and WO_x_ loading on the selective hydrogenolysis of glycerol to 1,3-PDO. A better 1,3-PDO yields (41% of anatase type Pt/W/Ti and 48% rutile type Pt/W/Ti) were obtained. It is found that the anatase-type Pt/W/A-Ti catalyst not only offer high catalytic activity with high 1,3-PDO yield (41%), but also exhibit excellent stability and can still maintain good catalytic activity after 5 cycles. However, the catalytic stability of rutile-type Pt/W/R-Ti is poor. Furthermore, for Pt/W/A-Ti catalysts, appropriate WO_x_ loading is also an important factor for the high catalytic selectivity of 1,3-PDO, and Pt/W/A-TiO_2_ with 5 wt% loading of WO_x_ achieved the best catalytic performance, owing to the suitable WO_x_ domains and high dispersion of W species. At last, the reaction mechanism of the catalytic reaction was studied by the *In-situ* DRIFT technique.

## Experimental

### Chemicals and materials

[(NH_4_)_6_(H_2_W_12_O_40_) nH_2_O], (aladdin, ≥ 99.5%), TiO_2_ (Rutile and Anatase, aladdin, ≥ 99.5%, Shanghai, China), Pt (NO_3_)_2_ (Xi’an Kaili new materials Co., Ltd., China, 0.15 g/ml Pt). All other chemicals and solvents (analytical grade) were purchased from Sinopharm Chemical Reagent Co., Ltd., China, SCRC.

### Catalyst preparation

All catalysts with different WO_x_ loadings were prepared using a step-wise impregnation method, and the loading of Pt was 2 wt% unless otherwise specified. Take the preparation of Pt/2W/R-TiO_2_ (R-TiO_2_ refers to rutile TiO_2_) as an example: Weigh 0.044 g of ammonium metatungstate (NH_4_)_6_(H_2_W_12_O_40_)·nH_2_O, (aladdin, ≥ 99.5%) into a 10 ml small beaker, add an appropriate amount of ultrapure water to dissolve, and weigh 1.92 g rutile-type TiO_2_ (aladdin, ≥ 99.5%) was slowly added to the above small beaker, stirred until the mixture was initially wet, and the mixture was dried in a 110°C oven for 12 h. After drying, the powder was poured into a crucible and calcined in a muffle furnace. The calcination temperature and time were 450°C, 4 h, respectively. The sample obtained after calcination was named W/R-TiO_2_. Then pipetting 133 μl of Pt (NO_3_)_2_ (0.15 g/ml Pt) solution into a 5 ml small beaker, add an appropriate amount of ultrapure water, weigh 0.98 g W/R-TiO_2_ and slowly add it to the small beaker After stirring to the initial wet state, it was placed in an oven and dried at 110°C for 12 h. After calcination, the target catalyst Pt/2W/R-TiO_2_ is finally obtained. For brevity, Pt/W/A-Ti and Pt/W/R-Ti are used to represent the anatase and rutile TiO_2_ supported Pt-W series catalysts, respectively. The loadings of Pt and W appearing in the text are calculated according to the mass of Pt and WO_3_, respectively.

### Catalyst characterization

The specific surface area of the catalysts and support were measured by a Quantachrome Quadrosorb evo apparatus. The XRD patterns of the catalysts were recorded on a Bruker D8A A25 X-ray diffractometer with Cu Kα radiation source (*λ* = 0.15406 nm). The voltage was 40 kV, and the tube current used was 40 mA. Ultraviolet-visible diffuse reflectance spectra (UV-vis DRS) were collected by a Cary 5000 UV-Vis-NIR spectrophotometer (Agilent, United States). The detector was a R928 PMT detector, the collection wavelength range was 200–800 nm, and the collection rate was 100 nm/min. The TEM and elemental mapping were obtained by a field emission electron microscope of the Talos F200S model from Thermo Fisher Scientific. The contents of W on the catalysts were determined using a Agilent 720/730 ICP-AES. The operating procedures of H_2_-TPD test, NH_3_-TPD test, CO-DRIFT absorption are shown in the experimental operation part of the Supplementary information.

*In-situ* DRIFT was performed on the same instrument for CO-DRIFT. The catalyst (50 mg) was milled and placed in the sample cell. Prior to the test, the catalyst was purged at 200°C for 1 h under He atmosphere, and the background was collected, and then reduced by introducing 10% H_2_/He for 1 h. After the reduction, switch to He, cool down to room temperature, invoke the background, add glycerol dropwise, and quickly heat up to 200°C, and collect the adsorption state spectrum. Switch to 10%H_2_/He to acquire reactive state spectra.

### Catalytic tests

A stainless autoclave reactor (with a lining capacity of 25 ml) was used for the hydrogenolysis of glycerol. For a typical run, 0.3 g of catalyst and 4.0 g of 10 wt% glycerol aqueous solution were added into the reactor, sealed and purged with H_2_ (3 MPa) for 4 times to exhaust the air in the autoclave, and then reacted at 6 MPa H_2_ and 140°C for 6 h with 700 rpm magnetic stirring. After the reaction, the solid-liquid mixture in the lining of the reactor was taken out and weighed, and then a certain amount of 1,4-butanediol was added as an internal standard. Then the mixture was centrifuged to remove the catalyst for product analysis. The liquid product was analyzed by an Agilent 7890B gas chromatograph equipped with an HP-INNO Wax column (30 m × 0.32 mm × 0.50 μm) and a flame ionization detector (FID). The conversion (Conv.) of glycerol and the selectivity (Sel.) of each product are defined as follows:

Conv. (%) = (moles of glycerol converted)/(moles of glycerol input) ×100% (1)
Sel. (%) = (moles of a specific product)/(moles of glycerol converted) ×100% (2)

## Results and discussion

### Effects of reaction conditions and impregnation order of Pt and WO_x_

**FIGURE 1:**
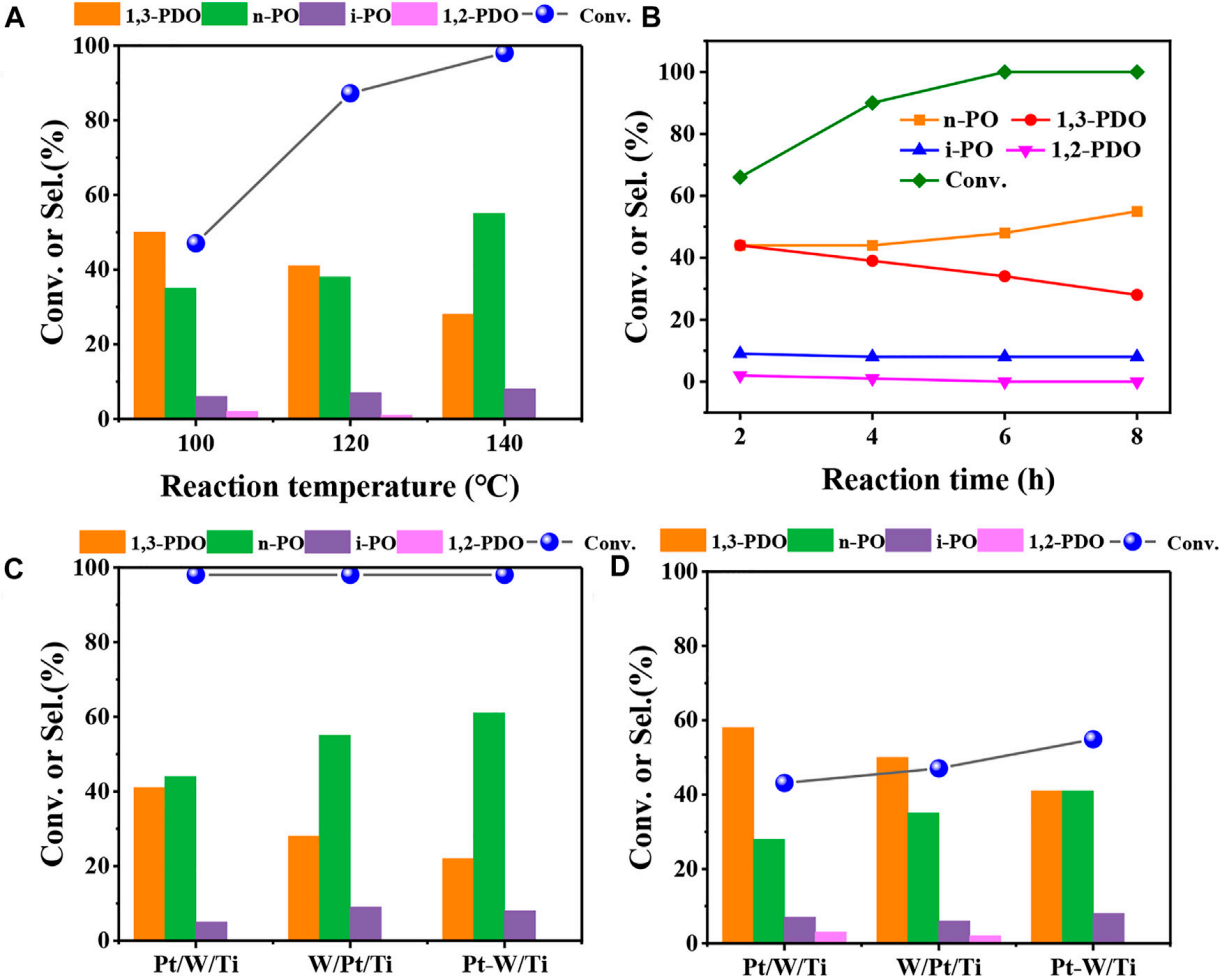
Catalytic results of glycerol hydrogenolysis at different reaction temperature **(A)** and different reaction time **(B)** over 5W/Pt/A-Ti catalyst; Catalytic results of glycerol hydrogenolysis over different catalysts at 140°C **(C)** and 100°C **(D)**. Reaction conditions: 0.3 g of catalyst (Support: A-Ti, WO_x_: 5wt%) and 4.0 g of 10 wt% glycerol aqueous solution, 6 MPa H_2_, **(B,C)** 140°C, 6 h (Pt/W/Ti means that W was loaded first, W/Pt/Ti means that Pt was loaded first and Pt-W/Ti means that Pt and W were loaded together).

The effect of reaction temperature was firstly investigated to determine the optimal reaction condition for glycerol hydrogenolysis with 5W/Pt/A-Ti as a probe catalyst. As the results shown in Figure 1A, it can be seen that the conversion of glycerol increased, while the selectivity of 1,3-PDO decreased with the increase of the reaction temperature. This result was in line with expectations because higher temperature speeds up the reaction rate for both glycerol hydrogenolysis to 1,3-PDO and further hydrogenolysis of 1,3-PDO to n-propanol (n-PO). Then, the reaction conducted for different reaction time at 140°C and 6 MPa shows that the conversion of glycerol reached 100% after reaction for 6 h (Figure 1B). It can be clearly seen that the selectivity of 1,3-PDO gradually declined and the selectivity of n-PO gradually increased as the reaction progress, while the selectivity of the other two by-products (1,2-PDO and i-PO) maintain almost unchanged. These results indicated that 1,3-PDO can further goes on hydrogenolysis to n-PO and therefore a proper reaction time is important.

We then explored the effects of impregnation order of Pt and WO_x_ on the glycerol hydrogenolysis and the results are displayed in Figures 1C,D. Here Pt/W/Ti means that W was loaded first, W/Pt/Ti means that Pt was loaded first and Pt-W/Ti means that Pt and W were loaded together. It can be seen that although all three catalysts achieved the full conversion of glycerol at 140°C, the selectivity of products were totally different. The Pt/W/Ti catalyst gave the highest yield (41%) of 1,3-PDO and Pt-W/Ti gave the lowest yield (21%) of 1,3-PDO. Since all three catalysts achieved the full conversion of glycerol and it is unreliable to compare the activity and selectivity at such a high conversion. We reduced the temperature to 100°C to lower the reaction rate and compare their activity and selectivity at a lower glycerol conversion (Figure 1D). It can be clearly seen that Pt/W/Ti offered the highest selectivity (58%) of 1,3-PDO, although the conversion of glycerol was slightly lower. Nevertheless, Pt/W/Ti catalyst gave the highest yield of 1,3-PDO. These results suggest that the impregnation order of Pt and WO_x_ has a great influence on the catalytic performance for glycerol hydrogenolysis, probably because different impregnation order of Pt and WO_x_ on TiO_2_ surface gives the different Pt-W interface.

### Effects of different crystal phases of TiO_2_

**FIGURE 2:**
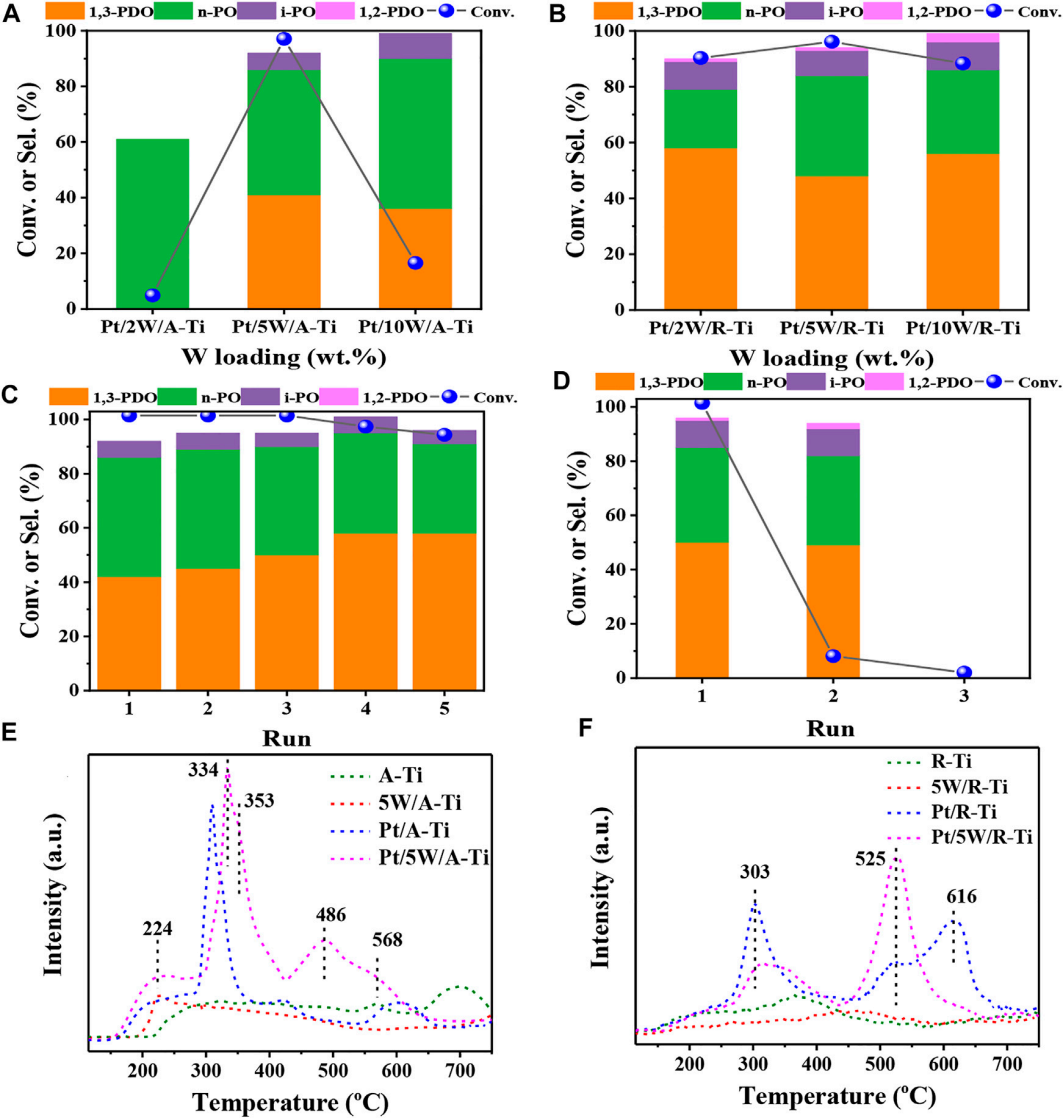
Comparative results obtained for the Pt/W/A-Ti **(A)** and Pt/W/R-Ti **(B)** fabricated under conditions of varying WO_x_ loadings. The cycle stability tests of Pt/5W/A-Ti **(C)** and Pt/5W/R-Ti **(D)**. NH_3_-TPD profiles recorded for the catalysts and support A-Ti **(E)** and R-Ti **(F)**. Reaction conditions: 0.3 g of catalyst and 4.0 g of 10 wt% glycerol aqueous solution, 6 MPa H_2_, 140°C, 6 h.

We then explored the effects of different crystal phases of TiO_2_ on the catalytic performance of glycerol hydrogenolysis. As shown in Figures 2A,B, we compared the catalytic performances of glycerol hydrogenolysis over two types of catalysts (i.e., Pt/W/A-Ti and Pt/W/R-Ti) with three different W loadings (i.e., 2%, 5%, and 10%). It is found that the initial activities of Pt/W/R-Ti catalysts were obviously better than the Pt/W/A-Ti catalysts for all 3 W loadings. Specifically, the yield of 1,3-PDO for Pt/5W/R-Ti (48%) was slightly higher than that for Pt/5W/A-Ti (41%) when the W loading was 5 wt%. However, the stability of Pt/5W/A-Ti catalyst was much better than that of the Pt/5W/R-Ti catalyst, as shown in Figures 2C,D, the Pt/5W/A-Ti maintain a very good catalytic activity after 5 cycles while Pt/5W/R-Ti almost completely lost its activity for the second run. The rapid deactivation of Pt/5W/R-Ti catalyst can be attributed to the severe leaching of the WO_x_ species, as we can visually see from Supplementary Figure S1 that the reaction solution became dark blue after reaction over Pt/5W/R-Ti, while the solution maintains colorless and transparent after reaction over Pt/5W/A-Ti. The severe leaching of WO_x_ species was also confirmed by ICP-AES measurements and results are shown in Supplementary Table S1. In addition, we also tried to change the durability of TiO_2_ (R-type) by changing the calcination temperature during catalyst preparation. However, as shown in Supplementary Figure S2, the reaction results show that increasing the calcination temperature in the catalyst preparation process leads to lower catalytic activity.

To further compare the difference between these two types of catalysts, NH_3_-TPD was used to investigate the acid strength and acidity of the two catalysts. Figure 2E reveals that the NH_3_-TPD profile of 5W/A-Ti sample presents a desorption peak at 224°C, corresponding to weak acid site. NH_3_-TPD profile of Pt/A-Ti presents two desorption peaks in the range of 300°C–350°C, corresponding to the medium acid sites (Zhou et al., 2020) (Zhou et al., 2019) (Zhao et al., 2021). Compared to the peaks corresponding to 5W/A-Ti and Pt/A-Ti, the desorption peaks corresponding to the Pt/5W/A-Ti catalysts shifted toward the high-temperature region, indicating that the interaction between Pt, W, and Ti increased the acid strength of the catalyst. The information present in the NH_3_-TPD profile of R-Ti type samples (Figure 2F) is different from the information present in A-Ti type samples (Figure 2E). The desorption peak at 616°C of Pt/R-Ti is shifted to 525°C in the profile recorded for Pt/5W/R-Ti. This can be potentially attributed to the interaction of Pt, W, and R-TiO_2_ in Pt/5W/R-Ti catalyst. However, it can be seen that the acid strength of Pt/5W/R-Ti is higher than that of Pt/5W/A-Ti, and this can explain the higher initial activity of Pt/5W/R-Ti for glycerol hydrogenlysis to 1,3-PDO (Feng et al., 2019).

In order to better compare the difference of total acid amount among different samples, we used the integral peak area ratio (the 5W/R-Ti sample with the smallest integral peak area was taken as the benchmark, and the integral peak area of the NH_3_-TPD curve desorption peak of other samples was compared with the integral peak area of the 5W/R-Ti sample) for semi-quantitative processing of the total acid content of each sample. The results were listed in Supplementary Table S2. Overall, the total acid content in A-Ti and the samples loaded with active components was higher than the total acid content recorded for the R-Ti counterparts. The effect of Pt on the acid content of the carrier is greater than the effect of W species, indicating that the presence of Pt results in the generation of additional acidic sites on the TiO_2_ support. From the acid content results of the two catalysts, it can be seen that the total acid amount of Pt/5W/A-Ti is higher than that of Pt/5W/R-Ti, but the initial yield of 1,3-PDO of Pt/5W/A-Ti is lower. This may suggest that only strong acid sites are responsible for glycerol hydrogenolysis of glycerol to 1,3-PDO. However, although the initial activity of Pt/5W/A-Ti is slightly lower, it has better application potential due to its ultra-high stability.

### Effects of WO_x_ loading and chemical state

**FIGURE 3:**
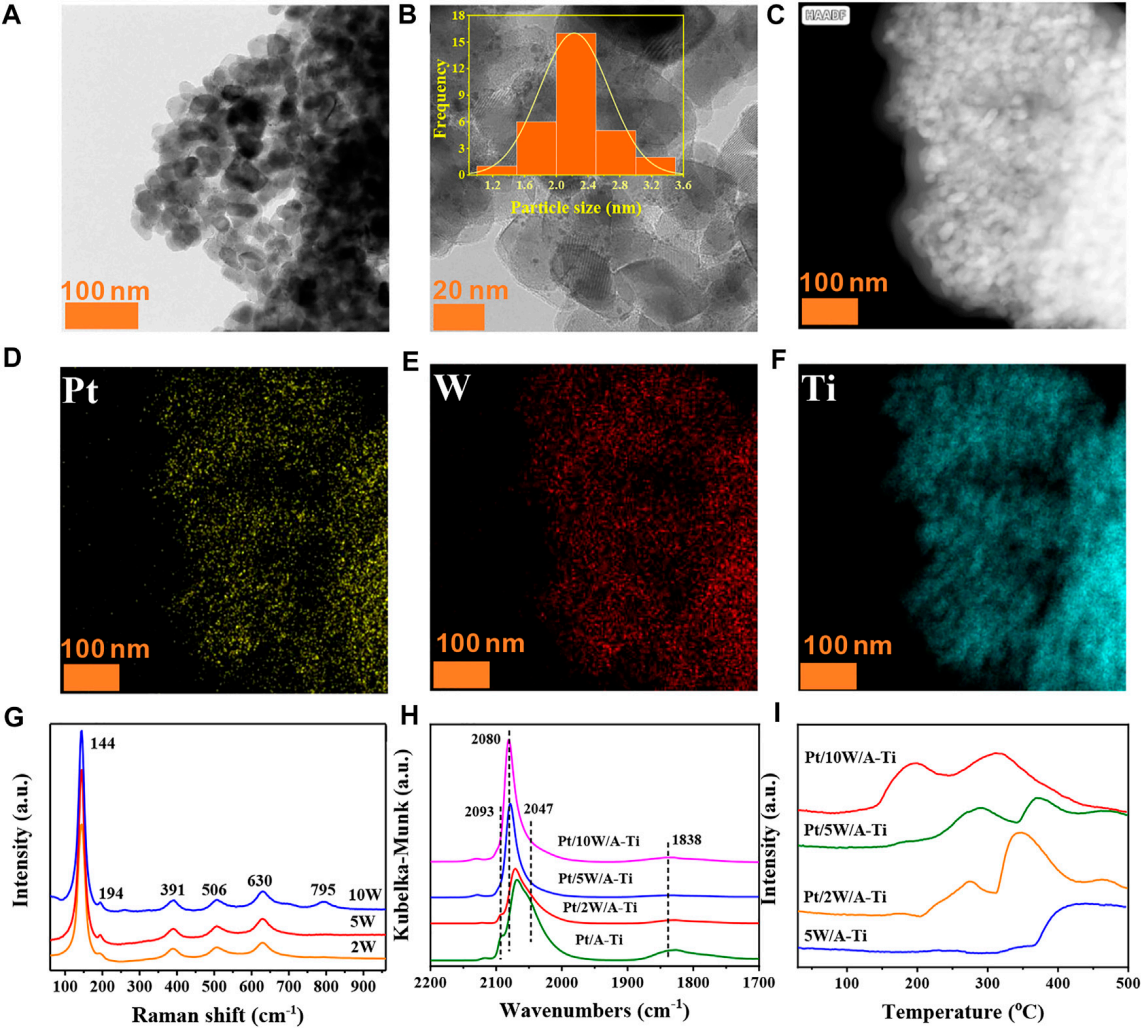
TEM image of the freshly reduced Pt/5W/A-Ti **(A,B)**, elemental mapping images of the freshly reduced Pt/5W/A-Ti **(C–F)**. Raman spectral profiles recorded for Pt/W/A-Ti **(G)**. DRIFTs of CO adsorption on the Pt/W/A-Ti catalysts prepared under conditions of varying W loadings **(H)**. H_2_-TPD curves recorded for the Pt/W/A-Ti series catalysts **(I)**.

The catalytic results of the catalysts indicated that the appropriate loading of WO_x_ species is a key factor for the catalytic performance. Therefore, we further investigated the loading of WO_x_ on the Pt/5W/A-Ti catalyst. From the TEM and elemental mapping images, it can be concluded that the distribution of Pt and WO_x_ on the Pt/5W/A-Ti catalyst is highly uniform (Figures 3A–F). Texture analysis of the catalysts revealed that with an increase of the W loading, the specific surface area and pore volume of the Pt/W/A-Ti catalyst decreased, while the mean pore size remained constant (Supplementary Table S3). This is attributed to that W species is primarily loaded onto the pores of the TiO_2_ support (García-Fernández et al., 2017). Raman spectroscopy was used to investigate the effect of W species on the catalyst. As shown in Figure 3G, the vibration peaks appearing at 144, 194, 391, 506, and 630 cm^−1^ were attributed to the anatase TiO_2_ units. The peaks appearing at 391, 506, and 630 cm^−1^ correspond to the symmetrical bending vibration, antisymmetric bending vibration, and symmetrical stretching vibration of the O-Ti-O unit on the TiO_2_ surface, respectively (Zhou et al., 2019) (He et al., 2018) (Xu et al., 2012) (Wachs, 2007) (Balta & Simsek, 2020) (Jia et al., 2021). Peak at 795 cm^−1^ was observed in the Raman spectral profile recorded for the Pt/10W/A-Ti catalyst. This peak can be attributed to the stretching vibration mode of W-O in bulk WO_3_. In general, when the W content in the catalyst is > 5 wt%, vibration peaks attributable to bulk WO_3_ are observed in the Raman spectral profiles recorded for the Pt/W/A-Ti catalysts. These results indicate that when the loading of W is higher than 5 wt%, bulk WO_3_ is formed, resulting in a decrease in the catalytic activity. This is consistent with the results from XRD pattern (Supplementary Figures S3A, B). However, decreasing the W laoding to 2 wt% as Pt/2W/A-Ti catalyst results in the lack of WOx active species. These results show that 5 wt% is the best WO_x_ loading for Pt/W/A-Ti catalyst, consistent with the catalytic reaction results shown in Figure 2A. Analysis of Supplementary Table S4 and Supplementary Figure S4 revealed that the W species in these three different W supported catalysts are likely to exist in the form of W_12_ clusters because their spectral information and E_g_ values are similar with ammonium metatungstate (AMT) which exists as W_12_ clusters structure (Zhao et al., 2021) (Yang et al., 2022) (Xu et al., 2021).

To further explore the effect of WO_x_ loading on the chemical state of Pt, we used CO as a probe molecule to characterize a series of catalysts with different W contents. As shown in Figure 3H, the peaks in the range of 2000–2,100 cm^−1^ are attributed to the linear adsorption of CO on the Pt site, and the peak at 1838 cm^−1^ is attributed to the bridge adsorption of CO on the metallic Pt unit (Xin et al., 2021) (Zhou et al., 2021) (Botao Qiao et al., 2011). The profiles recorded for Pt/A-Ti and Pt/2W/A-Ti reveal the presence of a shoulder peak at 2093 cm^−1^, which is attributed to the linear adsorption of CO on the surface of the PtO_x_ species. The molar ratio of CO to PtO_x_ is 1:1 in this case (Zhao et al., 2020). However, this peak was absent in the profiles recorded for the Pt/5W/A-Ti and Pt/10W/A-Ti samples, indicating that the PtO_x_ content in the catalysts fabricated under conditions of high WO_x_ loading was higher than the PtO_x_ content in the samples devoid of tungsten and fabricated under conditions of low WO_x_ content. The species are more easily reduced to Pt^0^. The peaks at 2047 cm^−1^ and 2080 cm^−1^ in Figure 3H are attributed to the bonding of CO with the coordinatively unsaturated and coordinatively saturated Pt sites, respectively. The former appears in the spectral lines of the Pt/A-Ti and Pt/2W/A-Ti samples. The results reveal that with an increase in the W loading, the number of unsaturated Pt sites in the catalyst decrease. This can be explained by the coordination effect between the coordination-unsaturated Pt and W, which results in the conversion of these sites to coordination saturated sites. In addition, the peaks corresponding to CO adsorption on the coordination saturated Pt sites blue-shifted with an increase in the W loading. This indicated electron transfer (Pt→W) between Pt and W (Yang et al., 2018). More importantly, the strength of the CO bridge adsorption peaks corresponding to the three W-loaded samples was weaker than the strength of the peaks corresponding to the W-free Pt/A-Ti units. This indicates that W does interact with Pt to cover Pt to a certain extent. The number of adjacent Pt sites decreases, and among the three samples containing W, the bridge adsorption peak in Pt/5W/A-Ti is observed to be the weakest. This proves our finding that Pt/5W/A-Ti is the best catalyst as it is characterized by the maximum area of the Pt/WO_x_ interface, which is consistent with the catalyst characterization and catalytic reaction results.

Figure 3I presents the H_2_-TPD diagram of the Pt/W/A-Ti series catalysts. Two desorption regions are observed in the H_2_-TPD curves recorded for the catalysts prepared when the W loadings were 2 wt%, 5 wt%, and 10 wt%. Unlike the case of the 5W/A-Ti sample devoid of Pt, the desorption peak at higher temperature regions can be attributed to W/Ti, and the desorption peak at the lower temperature regions can be attributed to Pt-H. The desorption peak temperature corresponding to Pt/5W/A-Ti is higher than that of the samples corresponding to 2 wt% W and 10 wt% W, indicating that the strength of adsorption of H species on the surface of Pt/5W/A-Ti is the strongest (Kuang et al., 2020) (He et al., 2018).

### Reaction mechanism study

**FIGURE 4:**
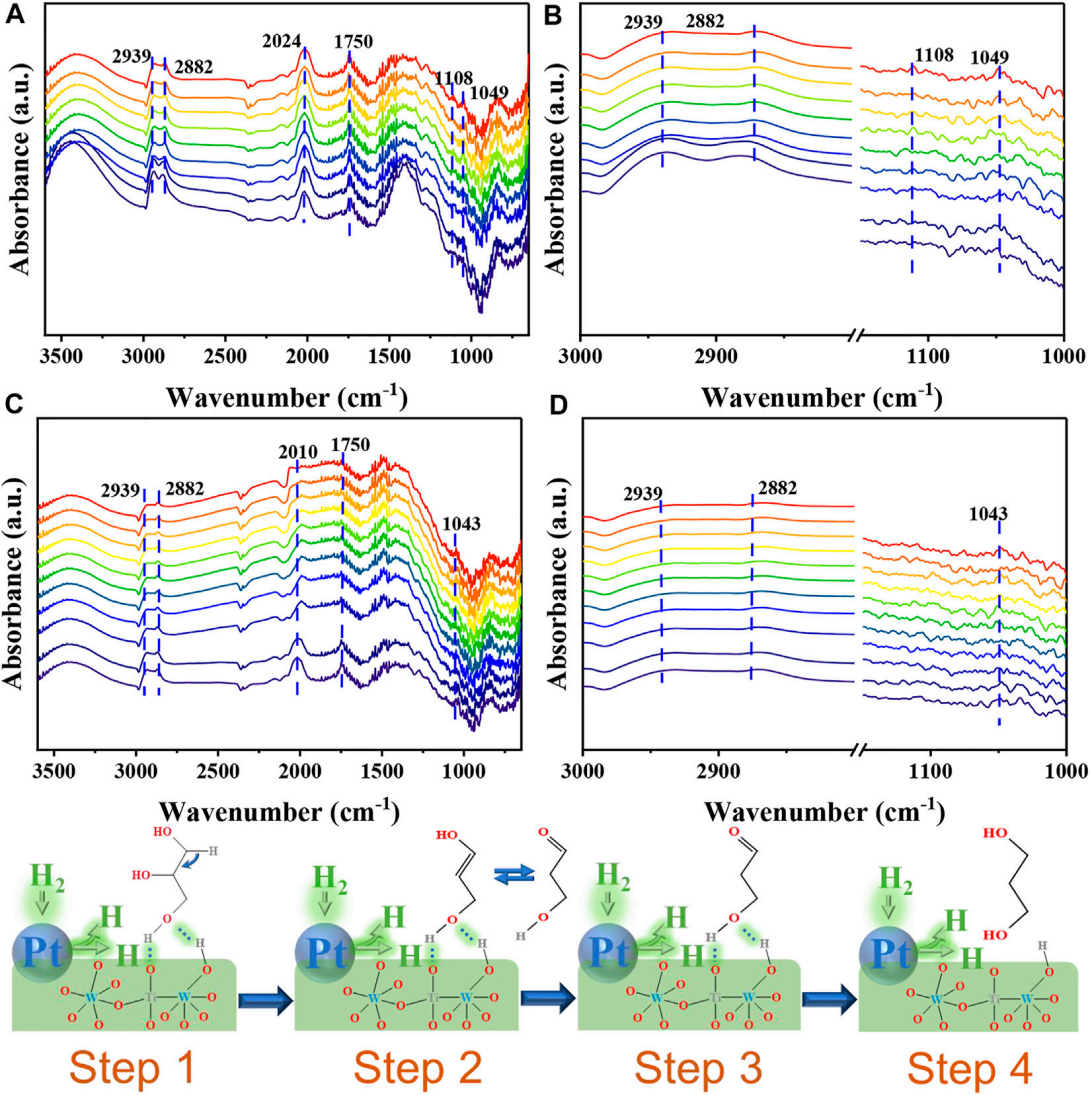
*In-situ* DRIFT profiles recorded for the adsorption of glycerol on Pt/5W/A-Ti studied within the investigated region **(A)**, and the adsorption state in the (C–H), 3,000–2,700 cm^−1^, and (C–O), 1,150–950 cm^−1^, regions **(B)**. Reactive state of glycerol on Pt/5W/A-Ti studied within the investigated region **(C)** and reactive state in the (C–H), 3,000–2,700 cm^−1^, and (C–O), 1,150–950 cm^−1^, regions **(D)**. Schematic diagram of the reaction mechanism (Step 1–4).

There are many studies on the mechanism of glycerol hydrogenolysis to produce 1,3-PDO (Cheng et al., 2021) (Feng et al., 2019) (García-Fernández et al., 2017). However, we traced the reaction path by *in-situ* DRIFT and found that glycerol was first converted to enol form and then further converted to 1,3-propanediol. As shown in Figure 4A which is the adsorption state infrared spectrum of glycerol, an absorption peak appears at 1750 cm^−1^, corresponding to the vibration of the C=O bond (García-Fernández et al., 2017), indicating that glycerol is converted to an enol-isomer when adsorbed on the catalyst which corresponds to step 1 and 2. Figures 4A–D all reveals the presence of two absorption peaks at 2,939 cm^−1^ and 2,882 cm^−1^. These peaks correspond to the symmetric, and asymmetric vibrations of the C-H units present in glycerol, respectively (Juan del Pozo et al., 2017). The peak appearing in the range of 2,400–2000 cm^−1^ may correspond to the absorption peak of CO_2_ (García-Fernández et al., 2017). Peaks were also detected at 1,108 cm^−1^ and 1,049 cm^−1^. These peaks corresponded to the (C-O) unit of the 2^◦^ and 1^◦^ OH groups in glycerol, respectively (JohnCopeland et al., 2013). Analysis of Figure 4C which is reaction state infrared spectrum of glycerol reveals that the peak at 1750 cm^−1^ is weak, indicating that enol glycerol is converted to 1,3-PDO following the passage of H_2_. The strength of the absorption peak observed in Figure 4D is less than the strength of the absorption peak at 1,108 cm^−1^ appearing in Figure 4B which corresponds to step 3 and 4. This indicated that glycerol was successfully converted to 1,3-PDO.

## Conclusion

In summary, Pt-WO_x_ catalysts supported on TiO_2_ with different crystal forms and WO_x_ loadings were prepared and studied for their catalytic performance in hydrogenolysis of glycerol. The Pt-WO_x_ catalysts supported by different crystalline phases of TiO_2_ exhibit completely different catalytic activities and catalytic stability. The Pt/W/A-Ti catalysts exhibit slightly lower initial catalytic activity than the Pt/W/R-Ti catalysts, but possess much higher stability owing to their excellent anti-leaching ability. Moreover, WO_x_ loading significantly influenced the catalytic performance and catalysts with moderate WO_x_ loading (5 wt%) exhibited the best catalytic performance. The results obtained using the CO DRIFT technique revealed the presence of special types of interactions between Pt and WO_x_. WO_x_ covers Pt to a certain extent, and 5 wt% loading of WO_x_ at the Pt/WO_x_ interface help achieve the maximum catalytic activity for glycerol hydrogenolysis. The reaction mechanism associated with the conversion of glycerol on Pt/5W/A-Ti was discussed using the *in-situ* DRIFT technique, and the reaction path for the conversion of glycerol to 1,3-PDO was proposed.

## Data Availability

The original contributions presented in the study are included in the article/Supplementary Material, further inquiries can be directed to the corresponding authors.
