# Supplementary material for: Hydrogenolysis of glycerol over TiO2-supported Pt-WOx catalysts: Effects of the TiO2 crystal phase and WOx loading
Source: Front Chem. 2022 Sep 21;10:1004925. doi: 10.3389/fchem.2022.1004925 (PMC9532750; doi:10.3389/fchem.2022.1004925)
Supplement: Supplementary file 1 [file DataSheet1.docx]

**Supplementary information**

**Hydrogenolysis of Glycerol over TiO_2_-Supported Pt-WO_x_ Catalysts: Effects of the TiO_2_ crystal phase and WO_x_ loading**

Yaju Wang^1,2^, Zhiming Zhou,^2^ Chao Wang^3,4^*, Leihong Zhao^1^, Qineng Xia^1,2^*

**1** Key Laboratory of the Ministry of Education for Advanced Catalysis Materials, Institute of Physical Chemistry, Zhejiang Normal University, Jinhua 321004 (P. R. China).

**2** College of Biological, Chemical Science and Engineering, Jiaxing University, Jiaxing 314001 (P. R. China).

**3** College of Chemical Engineering, Qingdao University of Science and Technology, Qingdao 266042, (P. R. China).

**4** Yankuang Technology Co., Ltd., Shandong Energy Group Co., Ltd., Jinan 250101, (P. R. China).

E-mail: [chao.wang@qdu.edu.cn](mailto:chao.wang@qdu.edu.cn); [xiaqineng159@163.com](mailto:xiaqineng159@163.com)

**Experimental (additional)**

The H_2_-TPD analysis was performed using a Quantachrome ChemBET Pulsar TPR/TPD apparatus equipped with a TCD. The specific operation steps are as follows: Weigh 0.2 g of the sample into a U-shaped quartz tube, add an appropriate amount of quartz wool to each end, and then load the quartz tube into the analysis station. First, reduce the sample with 10% H_2_/Ar mixture at 300 ℃ for 0.5 h, and then use the built-in fan in the instrument to cool down the sample. When the temperature is stable at about 30 ℃, continue to use 10% H_2_/Ar mixed gas atmosphere was maintained for 0.5 h to ensure that the adsorption of H_2_ on the catalyst surface was saturated, and then the gas path was switched to high-purity Ar. When the baseline was stable, the temperature-programmed desorption analysis was started, and the heating was increased to 500 ℃ at a heating rate of 20 ℃/min.

The NH_3_-TPD test was conducted on the same instrument for H_2_-TPD. The specific operation steps are as follows: Weigh 0.2 g of the sample into a U-shaped quartz tube, add an appropriate amount of quartz wool to each end, and then load the quartz tube into the analysis station. First purge the sample with He at 500 ℃ for 0.5 h, then use the built-in fan in the instrument to cool down the sample. When the temperature is stable at about 115 ℃, switch the gas to 10% NH_3_/N_2_ for 1 h to ensure that the sample has adsorbed ammonia to saturation. Then, the pipeline is purged with He to remove the NH_3_ molecules physically adsorbed in the pipeline and on the sample. After the baseline reached a steady state, ramp up to 750 °C at a ramp rate of 20 °C/min.

CO-DRIFT adsorption was performed on the Nicolet iS50 FTIR spectrometer with an MCT/A detector, with 64 scans and a resolution of 4 cm^-1^. The specific operation is as follows: Fill the bottom of the sample tank of the in-situ cell with an appropriate amount of quartz wool, place a copper mesh on it, take an appropriate amount of sample and add it to the copper mesh, and use the small tool equipped with the in-situ cell to press the added sample flat. The sample was first reduced in a 10% H_2_/Ar atmosphere at a temperature of 300 ℃ for 0.5 h, and then the entire pipeline was purged with He. The purpose was to remove H_2_ molecules adsorbed on the surface of the sample and H_2_ in the pipeline. After purging at 300 ℃ for 0.5 h, the temperature was lowered to 30 ℃ and the background stabilized, the background was collected and saved. Next, the gas was switched from He to 10% CO/He mixture. After 0.5 h, the pipeline was purged with He, and the sample signal was continuously collected during the purge period (the background needs to be deducted). When the signals are almost identical, end the signal acquisition and save the last acquired spectrum.


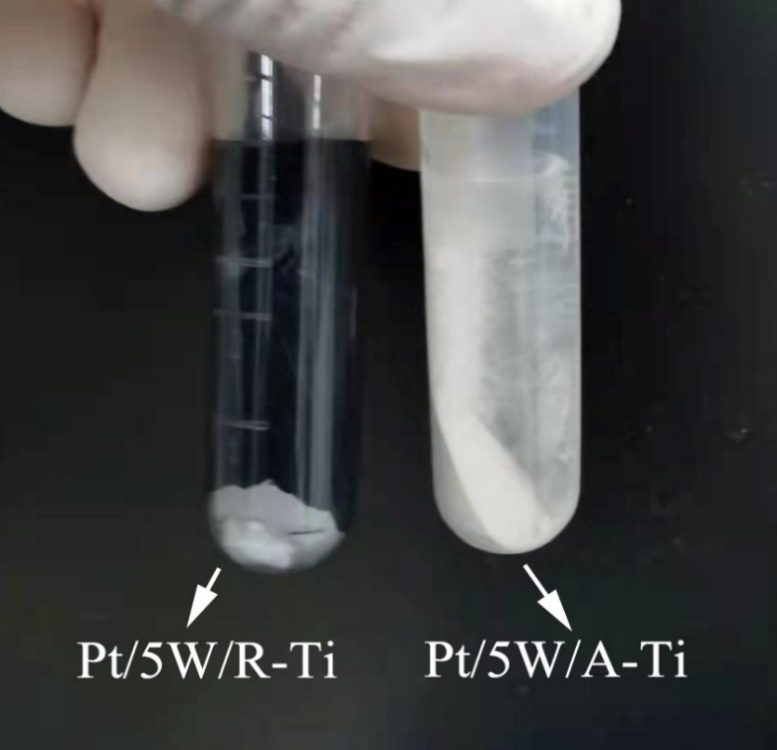


**Figure S1** The solid–liquid mixture obtained after the reaction was centrifuged.

Figure S2. The catalytic performances over Pt/5W/R-Ti calcined at different temperatures.

(a)

(b)

**Figure S3** XRD patterns recorded for Pt/W/A-Ti (a) and Pt/W/R-Ti (b) with different W loadings.

**Figure S4** UV-vis DRS spectral profiles recorded for Pt/W/A-Ti and the standard samples

**Table S1**. The ICP-AES results of the Pt/5W/R-Ti

| Sample | W loading (%) |
| --- | --- |
| Pt/5W/R-Ti | 3.89 |
| Pt/5W/R-Ti^a^ | 2.21 |

^a^ The catalyst recovered after reaction.

**Table S2** The NH_3_-TPD semi-quantitative results of the samples

| Sample | Ratio of the integrated peak area |
| --- | --- |
| R-Ti | 1.92 |
| 5W/R-Ti | 1.00 |
| Pt/R-Ti | 4.64 |
| Pt/5W/R-Ti | 4.71 |
| A-Ti | 6.36 |
| 5W/A-Ti | 6.93 |
| Pt/A-Ti | 7.57 |
| Pt/2W/A-Ti | 10.35 |
| Pt/5W/A-Ti | 15.08 |
| Pt/10W/A-Ti | 11.02 |

**Table S3** Physicochemical properties of the supports and the supported Pt/W/A-Ti catalysts.

| Sample | S_BET_^a^ (m^2^ g^-1^) | V_p_^a^ (cm^3^ g^-1^) | d_p_^a^ (nm) |
| --- | --- | --- | --- |
| A-Ti | 58 | 0.33 | 9.3 |
| Pt/A-Ti | / | / | / |
| Pt/2W/A-Ti | 55 | 0.32 | 9.3 |
| Pt/5W/A-Ti | 53 | 0.31 | 9.3 |
| Pt/10W/A-Ti | 48 | 0.27 | 9.3 |

^a^ Measured by N_2_ physisorption.

**Table S4** The fitting results of UV-vis DRS spectra.

| Sample | Fitting equation | R^2^ | E_g_ (eV) |
| --- | --- | --- | --- |
| WO_3_ | y = 1.923x - 4.629 | 0.99978 | 2.41 |
| AMT | y = 2.289x - 6.889 | 0.99992 | 3.01 |
| Na_2_WO_4_ | y = 2.573x - 12.12 | 0.99905 | 4.71 |
| Pt/2W/A-Ti | y = 1.959x - 5.863 | 0.99946 | 2.99 |
| Pt/5W/A-Ti | y = 2.049x - 6.207 | 0.99911 | 3.03 |
| Pt/10W/A-Ti | y = 1.976x - 5.932 | 0.99944 | 3.00 |
